# Functional and Morphological Changes in the Deep Lumbar Multifidus Using Electromyography and Ultrasound

**DOI:** 10.1038/s41598-018-24550-5

**Published:** 2018-04-25

**Authors:** Shanshan Zhang, Yi Xu, Xiulan Han, Wen Wu, Yan Tang, Chuhuai Wang

**Affiliations:** 1 Department of Rehabilitation Medicine, The First Affiliated Hospital, Sun Yat-sen University, Guangzhou, 510080 PR China; 2 Department of Rehabilitation Medicine, Zhujiang Hospital, Southern Medical University, Guangzhou, 510282 PR China

## Abstract

Surface electromyography (sEMG) studies have indicated that chronic low back pain (cLBP) involves altered electromyographic activity and morphological structure of the lumbar multifidus (LM) beyond pain perception; however, most studies have evaluated the superficial lumbar multifidus. It is difficult to record electromyography (EMG) signals from the deep multifidus (DM) to determine the neuromuscular activation patterns, making it difficult to determine the relationship between functional and structural changes in cLBP. We developed a novel method to record intramuscular EMG signals in the DM based on the sEMG system and fine-wire electrodes. We measured EMG signals of the DM in 24 cLBP patients and 26 pain-free healthy controls to identify changes in neuromuscular activation. We also used ultrasound to measure DM muscle thickness, cross-sectional area, and contraction activity to identify potential relationships between EMG activity and structural damage. cLBP patients had decreased average EMG and root mean square, but increased median frequency and mean power frequency. Average EMG was positively correlated with contractile activity, but not statistically correlated with noncontractile anatomical abnormalities. Our results suggest that cLBP alters the neuromuscular activation patterns and morphological structure of the contractile activity of the DM, providing insights into the mechanisms underlying pain perception.

## Introduction

The lumbar multifidus (LM) muscle plays an important role in stabilization during functional movements, and it accounts for two-thirds of lower lumbar segmental stability^1^. The deep fibers of lumbar multifidus (DM) act as a vital segmental stabilizer that allows for lateral flexion, rotation, and extension, while the superficial fibers are thought to contribute to spinal compression and extension. Moseley *et al*.^2^ assessed the timing of LM activation and demonstrated that the deep and superficial fibers were differentially activated. The electromyography (EMG) in the DM increased two- to three-fold and the latency of the loading response decreased during self-initiated perturbations. In contrast, increased EMG in the superficial fibers was not observed and the latency of the loading response increased^2^. Intersegmental deep muscle fibers are reported to play a substantial role in neuromuscular function to control the motion segment^3^.

Surface electromyography (sEMG) records muscle electrical activity to provide dynamic real-time measurement of neuromuscular function using surface electrodes. Extensive research has shown that chronic low back pain (cLBP) alters electromyographic activity of the LM in response to persistent nociceptive stimuli^4,5^. Dankaerts *et al*.^6^ used sEMG to record EMG signals from the superficial trunk muscles to investigate altered patterns of activation during nonspecific cLBP patients’ unsupported “usual” and “slumped” sitting. They found that the active extension pattern group exhibited higher levels of co-contraction of the superficial fibers of LM, iliocostalis lumborum pars thoracis, and transverse fibers of the internal oblique. Recent reports of EMG suggest that cLBP is related to altered control of the LM with delayed anticipatory activation prior to rapid limb movements when standing^4,5^. Deep fibers form the main structural component of the LM^2^. Although changes in EMG activity of the superficial fibers has enhanced our understanding of the pathogenesis of cLBP^5^, altered activity in the superficial fibers offers limited prognostic value. Less is known about whether the magnitude of DM activation changes in the presence of pain. However, it is difficult to record EMG signals in the DM using sEMG and surface electrodes to determine the neuromuscular activation patterns. Current research has focused on whether altered activity of the DM contributes to cLBP. Therefore, a method is needed to detect EMG signals in the DM to define their function.

The intramuscular fine-wire electrode modeling approach was developed as a useful method to detect EMG signals from targeted muscles during isometric contraction^7,8^. This technique has proved to be an objective assessment method for evaluating neuromuscular activities of the deep muscle (i.e., brachialis muscle, DM, and transversus abdominis) in asymptomatic subjects and stroke patients^7–9^. However, it does not effectively measure maximal muscle strength and intrinsic muscle contractile properties due to low levels of electrical activity. Integrating advantages of the sEMG system and fine-wire electrode method, we present a novel method to record EMG signals and identify changes in neuromuscular function of the DM in cLBP. A better understanding of how pain effects the DM activity can help explain deep back muscles dysfunction commonly encountered clinically.

Recent attempts to understand cLBP have focused on the interaction between functional and structural changes, including central motor reorganization^4,10^, and atrophy of the DM has been identified as a potentially important biological process. A retrospective study reported bilateral and generalized DM atrophy in patients with cLBP seen on magnetic resonance images (MRI)^11^. Patients with cLBP showed a significant reduction in DM resting thickness^12^, cross-sectional area^13^, and lower percentage thickness change during activation with a contralateral arm lift^14^. Also, induced-pain studies using ultrasound to assess DM function showed altered muscle activity during voluntary and automatic movements^15–17^. However, ultrasound or MRI are less likely than intramuscular EMG to accurately assess DM activity patterns, and, to our knowledge, no previous studies have investigated the relationship between changes in EMG activity and morphological features of the DM. Therefore, we chose ultrasonography in our study to assess DM thickness, cross-sectional area, and contraction activity to investigate a potential link between EMG activity and structural changes in cLBP.

The aim of our study was to determine how cLBP impacts neuromuscular activation of the DM during functional movement with maximum voluntary isometric contraction (MVIC). This study provides a better understanding of profound alterations in the intrinsic contractile properties and motor coordination of the DM in cLBP. Specifically, our objectives were: (1) to identify the time-frequency features of EMG signals of the DM; and (2) to investigate DM thickness and cross-sectional area at rest, and thickness change during MVIC; and (3) to explore potential relationships between EMG activity and contractile behavior of DM during functional movement in the presence of LBP. We hypothesised that changes in neuromuscular activation of lumbar DM should be evident in cLBP. Also, we hypothesized that decreased muscle thickness and contraction amplitude of the DM are associated with lower muscle strength in patients with LBP, and that muscle atrophy is a contributing factor to predict future LBP.

## Methods

### Participants

Twenty-four patients with cLBP were recruited from inpatient and outpatient departments, which were diagnosed by a physician in the First Affiliated Hospital of Sun Yat-sen University. Inclusion criteria included: (1) history of cLBP for >3 months; (2) current pain intensity assessed by the 10-cm visual analogue scale (VAS) with a score ranging from 3–6; (3) body mass index (BMI) within ±20% of international standards; and (4) right-handed. Exclusion criteria included: previous lumbar spinal surgery, presence of ankylosing spondylitis, systemic disease, and severe neurological or psychiatric disorders. Potential female participants who were pregnant or suffered from dysmenorrhea were also excluded. The Oswestry Disability Index was used to assess pain-related disability.

The control group consisted of 26 healthy participants with no self-reported symptoms or history of activity-limiting LBP, which were recruited from posted notices to students on campus and staff employed at the hospital. Group characteristics are presented in Table 1. There were no group differences for gender, age, height, weight, BMI or educational level. This study was approved by the Institutional Research Ethics Committee of the First Affiliated Hospital of Sun Yat-sen University (Guangzhou). All participants reviewed and signed the informed consent sheet. We confirm that all methods were performed in accordance with the guidelines and regulations of our ethical statement.

**Table 1:** Characteristics of participants (mean ± SD).

	cLBP	healthy control	*P*-value
Participants (*n*)	24	26	
Gender (M:F)	11:13	13:13	
Age (years)	35.86 ± 7.64	32.35 ± 7.19	0.10
education level (years)	12.96 ± 2.46	13.69 ± 1.89	0.25
Height (cm)	163.00 ± 6.69	165.23 ± 7.35	0.27
Weight (kg)	58.08 ± 8.57	57.31 ± 9.13	0.76
BMI (kg/m^2^)	21.75 ± 2.05	20.97 ± 2.02	0.19
Pain intensity (VAS)	3.96 ± 1.04	—	
Pain duration (years)	6.83 ± 6.12	—	
ODI (%)	32.75 ± 20.06	—	

cLBP, chronic low back pain; BMI, body mass index; ODI, Oswestry Disability Index;

VAS, visual analog scale.

### Data acquisition

Each participant underwent rest and maximum isometric contraction measurements of EMG signals of the DM using the sEMG system, and muscle thickness and cross-sectional area measurement using ultrasound (SonoSite M-Turbo, Seattle, USA). Participants underwent measurements in randomly-assigned order, and the assessment lasted approximately 40 mins. Participants were told to maintain back muscle contractions during the task, and each subject was familiarized with MVIC of the DM during Biering-Sorensen testing. A single practice was performed prior to measurements being recorded.

#### EMG activity of the DM

To study intramuscular EMG activity of the DM, fine-wire electrodes (0.16 mm, Friendship Medical Electrodes Company, Xi’an, China) were fabricated from a pair of enamel-coated 12-cm wires which were inserted into a hypodermic needle (25 gauge, 60 mm length). We removed the enamel coating on the wires from 5 mm of the tip and 20 mm of the end of the wires to permit electrical conduction. After the needle and wires were sterilized, the fine wires were manipulated to slightly stagger their alignment by bending the tips back 3–5 mm prior to insertion.

With each participant relaxed in the prone position on the plinth, and with their head centered on midline, the fourth lumbar (L4) spinous process was marked for orientation using the iliac crests as reference points for the L4 vertebral level. Two recording electrodes in the DM were placed 2 cm lateral to the L4 spinous process on each side with an inter-electrode distance of 0.5–1 cm parallel to the longitudinal rotational axis of the spine. A reference electrode was placed 4–5 cm lateral to the recording electrodes. Prior to testing, compounded lidocaine cream was placed on the planned puncture sites for 5 min to reduce needle insertion pain.

**Figure 1:**
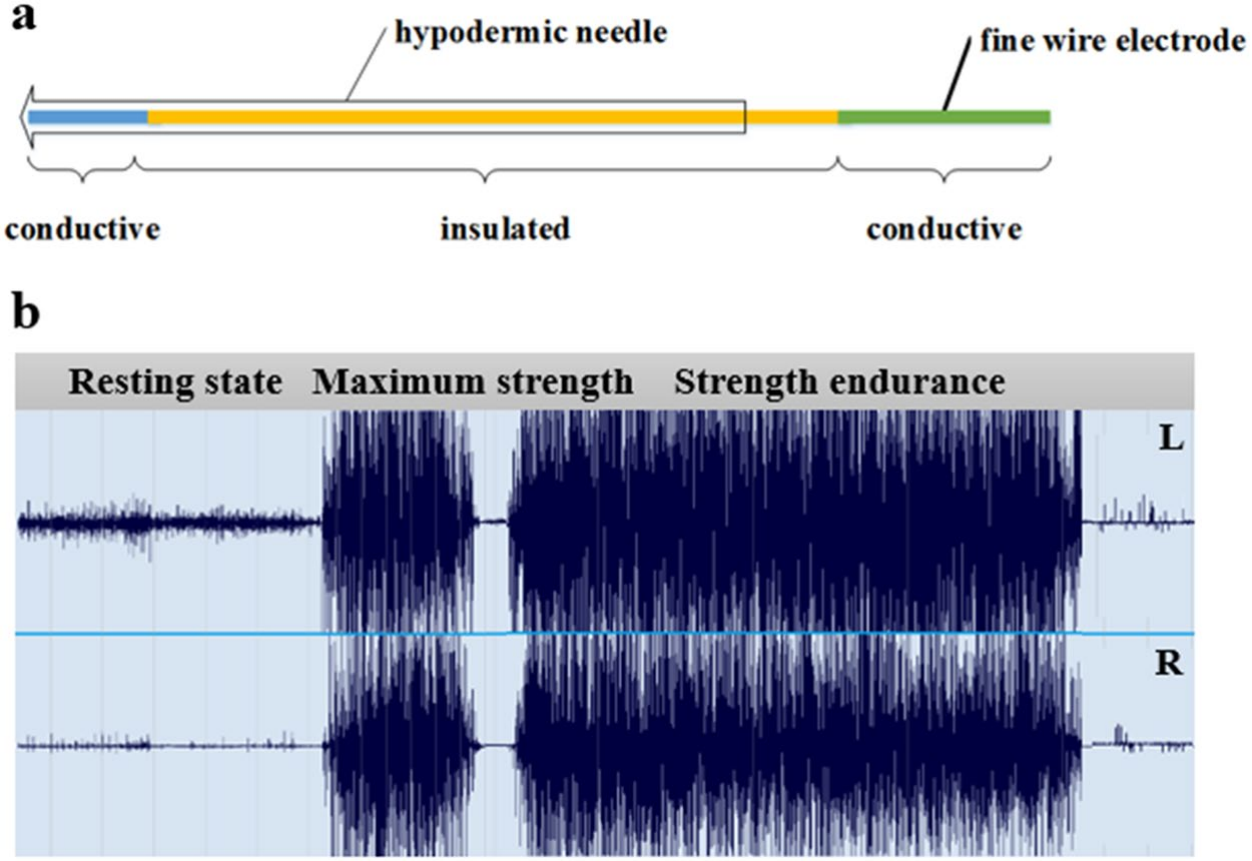
Intramuscular EMG activity of the deep multifidus (DM) muscle. (A) Maps of the fine-wire electrodes to record intramuscular EMG activity of the DM. A fine-wire electrode was inserted into (**a**) hypodermic needle. The enamel coating was removed from 5 mm of the electrode tip and 20 mm from the end for electrical conduction. (**b**) Representative EMG signals from the DM for each side during the resting state, maximum strength, and strength endurance. EMG, electromyography.

Four fine-wire electrodes were inserted perpendicularly deep into bilateral DM muscles until the hypodermic needle touched the lamina (Fig. 1a). The location of the recording electrodes was verified using ultrasound. The needles were then withdrawn, leaving the wire electrodes in the deepest portion of the DM. External fine wires were attached to the skin on each participant’s back using hypoallergenic medical tape, and the bare end of the electrodes were connected to amplifiers with alligator clip electrodes (Qiaotian Technology, Shenzhen, China). Two reference electrodes consisting of 7-cm wires inserted into hypodermic needles (24 gauge, 30 mm length) were inserted 2-cm deep into the back muscles. Needle-insertion pain subsided to pre-injection levels within approximately 20 s in each patient.

Data on bilateral DM activity was collected using a UMI-sEMG-I system (Umedstrr, Shaoxing, China) with a bandwidth of 15–1000 Hz, resolution of 0.1 μV, common mode rejection ratio of 110 db, and a low noise < 1 μV. Signals from the sEMG system were sampled at 3000 Hz and stored on the computer. Each participant underwent separate measurements to obtain EMG signals in the resting state and during MVIC of the DM. To obtain the MVIC data, participants lay in the prone position on a standard plinth with the upper limbs positioned overhead, elbows flexed to approximately 90°, and shoulders abducted to approximately 120°. Participants then lifted their head, trunk, and upper extremities with maximum effort. This position has been used previously to assess activation of the LM^14,18^. Verbal encouragement was given to the subjects with cLBP during MVIC.

All EMG signals were obtained using the following steps: (1) resting state: Participants were asked to keep their muscles relaxed, and EMG data were collected for 30 s; (2) maximum strength: Following the resting data collection, participants were asked to perform MVIC of the DM for 5 s, executed three times with a 30-s rest between movements. Typically, the mean value of the three repetitions was calculated for each subject. (3) strength endurance: Following a 60-s rest, participants were asked to maintain the same movement as in step 2 (MVIC of the DM) for 30 s (Fig. 1b). When the tests were completed and with participants still on the plinth, they were asked to rate perceived pain intensity during needle insertions and muscle contractions using the VAS.

#### Morphological changes in the DM

The morphological outcomes measured in this study were DM resting thickness, cross-sectional area, and thickness during MVIC using ultrasound with a high-frequency 6–13 MHz transducer. All scans were performed by one doctor educated to MD level who had clinical experience with real-time ultrasound of the musculoskeletal system. Assessments for DM measurements were recorded at L4 levels in two conditions: at rest and during MVIC.

Each subject lay prone on a standard plinth, and with their head centered on midline. The ultrasound transducer with electrically-conductive adhesive hydrogels was placed transversely and centrally on the L4 spinous processes then moved laterally until the spinous processes almost disappeared in the transducer light to obtain the maximum cross-sectional area of the DM without compressing the skin. Once a clear image of the DM was obtained, it was frozen on the screen and saved on the ultrasound scanner for measurement. The inferior border of the erector spinae muscle was used as a starting point and the superior tip of the facet joints as the second landmark for DM thickness measurement.

Three images on bilateral DM were taken at rest to measure thickness and cross-sectional area using the screen calipers. Next, subjects were asked to perform MVIC of the DM as detailed for the sEMG measurements, for 3–5 s. Once a clear image of the DM was obtained, it was frozen on the screen and saved on the scanner for measurement of DM thickness during MVIC using the screen calipers. A total of six bilateral DM images were captured at the L4 level.

### Data processing

EMG data were processed using sEMG analysis-feedback instrument system software V1.0 (Umedstrr, Shaoxing, China). A fast Fourier transform (FFT) was performed for spectrum analysis, and multiple features of the EMG signals were extracted during the resting state and MVIC tasks including the average EMG (AEMG), root mean square (RMS), median frequency (MF), and mean power frequency (MPF). Median frequency slope (MFs) and mean power frequency slope (MPFs) were also analyzed in each group.

### Statistical analysis

Statistical analysis was performed using SPSS 13.0 (SPSS Inc. Chicago, IL, USA). Descriptive statistics (mean ± SD) were calculated for age, education, BMI, pain intensity and pain duration. For between-group analyses, the respective mean data for both sides were used for each group. To compare changes in DM neuromuscular activation patterns, two-sample t-test was used to determine potential EMG signal differences for AEMG, RMS, MF, and MPF between groups. The paired t-test was used to compare within-subjects’ effects to examine the effect of chronic pain on DM muscle activity,

Morphological features were also evaluated with the two-sample t-test to identify significant differences in resting thickness, cross-sectional area, and thickness during MVIC. For the MVIC task, percentage thickness change was calculated as follows: [percentage thickness change = (contraction − rest)/rest × 100%]. The paired t-test was used to evaluate within-subject differences to examine the effect of persistent pain stimulation on the DM.

For each subject, Pearson’s correlation analysis was performed to determine potential correlations between EMG activity (AEMG) and morphological variables (resting thickness, cross-sectional area, thickness during MVIC) of the DM in the presence of cLBP. To observe whether neuromuscular activation or morphological changes of the DM varied with pain stimuli, correlation analysis was used to compare AEMG and resting thickness in cLBP using VAS score or pain duration. The level of statistical significance was set at *P* < 0.05.

### Data availability

The data that support the findings of this study are available from the corresponding authors on request.

## Results

A total of 50 participants (24 cLBP patients and 26 healthy controls) completed the study. All participants cooperated and none discontinued participation in the study because of unbearable pain. Data for each subject was included in all analyses.

All subjects experienced mild to moderate pain during needle insertions and muscle contraction. The cLBP group showed a higher pain intensity (average VAS: 3.88 ± 1.26) during needle insertions than did healthy controls (average VAS: 2.46 ± 0.81) (t = −4.75, *P* < 0.001). The average maximum pain score during muscle contraction in the cLBP group was 3.38 ± 1.66, which was higher compared with healthy controls (0.64 ± 0.46) (t = −8.04, *P* < 0.001). When the scans were completed, electrode-induced pain or foreign body sensation was relieved immediately after removing the wires and subsided to pre-insertion levels within 1–2 min.

### Between-group analyses of EMG activity

Compared with the matched healthy controls, cLBP patients showed a trend towards higher magnitude of activity in the resting state (t = 2.60, *P* = 0.01). Within-subject EMG analyses for cLBP patients demonstrated significantly higher magnitude of activity in the DM on the painful side compared with the nonpainful side (t = 2.63, *P* = 0.01).

During MVIC of the DM, cLBP patients showed decreased AEMG and RMS of the time domain, but increased MF and MPF of the frequency domain in the EMG measurements (*P* < 0.05, Table 2). There were also statistically significant differences between the two groups in MFs and MPFs, where the cLBP group had higher values than did the healthy control group (*P* < 0.05, Table 2). Figure 1b shows the EMG signals recorded from the DM for each side during each state (resting state, maximum strength, and strength endurance).

**Table 2:** Between-group analyses of EMG activity in deep multifidus muscle.

	cLBP	healthy control	t-value	*P*-value
AEMG	426.94 ± 146.41	771.44 ± 149.04	8.24	<0.001
RMS	267.79 ± 88.10	339.34 ± 71.72	3.16	0.003
MF	187.71 ± 29.67	169.13 ± 26.30	−2.27	0.024
MPF	200.00 ± 26.20	184.63 ± 22.51	−2.16	0.032
MFs	0.06 ± 0.06	0.17 ± 0.13	3.55	0.001
MPFs	0.05 ± 0.05	0.14 ± 0.10	3.58	0.001

cLBP, chronic low back pain; AEMG, average EMG; RMS, root mean square; MF, median frequency; MPF, mean power frequency; MFs, median frequency slope; MPFs, mean power frequency slope.

### Within-subject analyses of EMG activity in the DM in the cLBP group

Within-subject EMG analyses demonstrated that MF and MPF for the DM increased significantly in the cLBP group (t = 3.39/3.49, *P* < 0.01). However, no differences were observed in AEMG or RMS when comparing the painful and nonpainful sides (t = −1.35/−0.54, *P* > 0.05) (Table 3).

**Table 3:** Within-subject’s EMG activity of deep multifidus muscle in cLBP.

	Painful side	Nonpainful side	t-value	*P*-value
AEMG	389.97 ± 186.65	463.92 ± 209.65	−1.35	0.19
RMS	276.49 ± 143.99	259.09 ± 84.32	−0.54	0.592
MF	199.33 ± 34.48	176.10 ± 32.66	3.39	0.003
MPF	210.10 ± 29.60	189.90 ± 29.13	3.49	0.002

cLBP, chronic low back pain; AEMG, average EMG; RMS, root mean square; MF, median frequency; MPF, mean power frequency.

### Between-group analyses of morphological features

**Figure 2:**
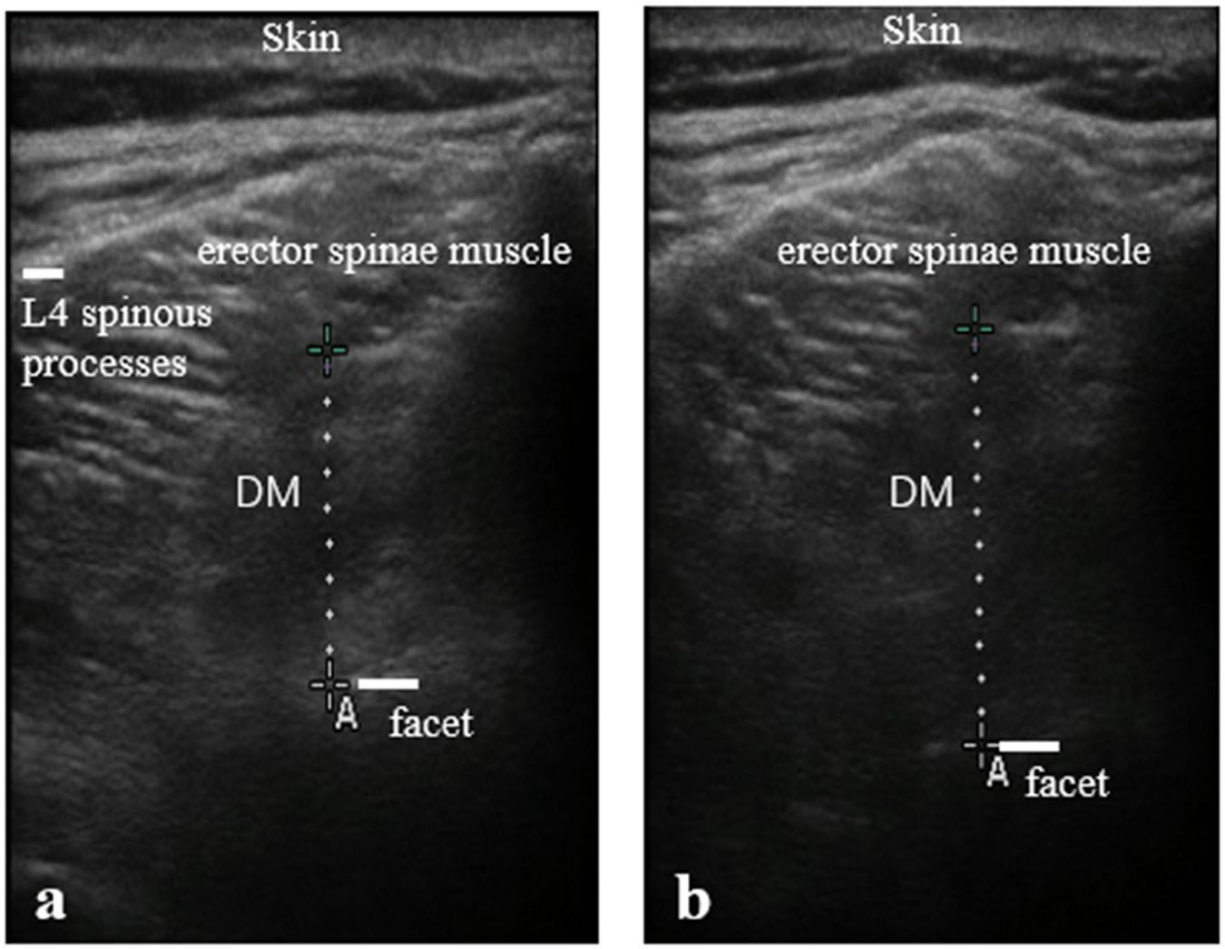
Representative ultrasound images of the deep multifidus (DM) from a patient with chronic low back pain (sagittal plane at L4 level) at rest (**a**) and during isometric activation (**b**). The line segments for labeling denotes the measure between the inferior border of the erector spinae muscle and the superior tip of the facet joints. L4, fourth lumbar vertebra.

We saw significant differences between groups for resting DM thickness and cross-sectional area (*P* < 0.001, Fig. 2). There were also statistically significant differences between groups in DM activation during MVIC, where the cLBP group demonstrated greater thickness decreases, and achieved a small effect size (*P* < 0.001). There were also statistically significant differences in the percentage thickness change during MVIC between groups (*P* < 0.001) (Table 4).

**Table 4:** Between-group analysis of Morphologic features in deep multifidus muscle.

	cLBP	healthy control	t-value	*P*-value
Resting thickness	1.66 ± 0.21	2.10 ± 0.20	7.58	<0.001
Thickness during MIVC	2.17 ± 0.34	2.95 ± 0.25	9.21	<0.001
Thickness change (%)	29.69 ± 8.62	40.43 ± 5.83	5.12	<0.001
Cross-sectional area	2.83 ± 0.74	4.36 ± 0.58	8.19	<0.001

cLBP, chronic low back pain; MVIC, maximum voluntary isometric contraction.

### Within-subject analyses of morphological features of the DM in the cLBP group

When comparing painful versus nonpainful sides, there was no significant difference in deep LM resting thickness, cross-sectional area, thickness during MVIC, or percentage thickness change within the cLBP group (*P* > 0.05, Table 5).

**Table 5:** Within-subject analysis of morphologic features in deep multifidus muscle in cLBP.

	Painful side	Nonpainful side	t-value	*P*-value
Resting thickness	1.66 ± 0.25	1.67 ± 0.21	−0.23	0.82
Thickness during MIVC	2.17 ± 0.37	2.16 ± 0.36	0.15	0.89
Thickness change (%)	30.79 ± 9.10	29.36 ± 12.78	0.51	0.62
Cross-sectional area	2.81 ± 0.81	2.85 ± 0.74	−0.4	0.69

cLBP, chronic low back pain; MVIC, maximum voluntary isometric contraction.

### Correlations between EMG activity and morphological variables in the DM

AEMG in the cLBP group was positively correlated with thickness during MVIC and percentage thickness changes (r = 0.47/0.50, *P* < 0.05), but not correlated with either DM resting thickness or cross-sectional area (r = 0.33/0.34, *P* > 0.05).

**Figure 3:**
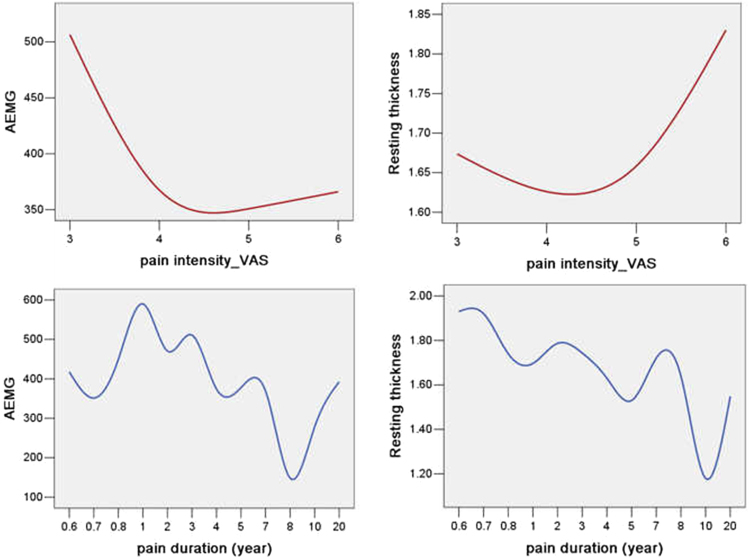
Correlation between functional and morphological changes in the deep multifidus (DM) and behavioral data in patients with chronic low back pain. (**a**,**b**) Distribution of average EMG values (**a**) and resting thickness measurements (**b**) of the DM for pain intensity (visual analog scale scores). (**c**,**d**) Distribution of average EMG (**c**) and resting thickness (**d**) of the DM for pain duration. EMG, electromyography.

AEMG was negatively correlated with VAS scores (r = −0.45, *P* = 0.03), but not correlated with pain duration (r = −0.28, *P* = 0.18). The DM resting thickness was negatively correlated with pain duration (r = −0.49, *P* = 0.02), but not correlated with pain intensity measured as VAS score (r = 0.11, *P* = 0.62) (Fig. 3).

## Discussion

In this study, we developed a novel method using the sEMG system and fine-wire electrodes to detect EMG signals and identify changes in neuromuscular activation of the DM in cLBP patients. Ultrasound-measured muscle thickness, cross-sectional area and contraction activity of the DM were also evaluated to identify a relationship between EMG activity and morphological features during persistent pain and to investigate the mechanism underlying the pathogenesis and pathophysiology of cLBP. Time-frequency analysis of EMG signals recorded from the DM showed that AEMG and RMS decreased significantly, but MF and MPF increased during MVIC in cLBP patients versus controls. Also, the cLBP group showed reduced thickness and cross-sectional area of the DM and achieved a small effect size. AEMG in the cLBP group was significantly positively correlated with DM thickness during MVIC and percentage thickness changes, but not correlated with either resting thickness or cross-sectional area, demonstrating that pain alters the magnitude of EMG activation of the DM during isometric back extensions.

Studies have shown a decrease in timing and amplitude of EMG activity and delayed activation of the DM in patients with cLBP^19,20^. AEMG and RMS were significantly decreased in our cLBP group, suggesting that recruitment of the DM is changed during MVIC, which potentially reduces its effectiveness during functional tasks^21^. Although the mean amplitude of activation changed significantly in the cLBP group, we found no differences between painful and nonpainful sides when subjects performed maximum isometric back extensions. These results differed from most previous studies showing that EMG activity in the DM had a relatively lower magnitude on the painful side. Our results are supported by other researchers who suggest that the effect of pain on muscle activation of the DM relates to the type of activity being performed^7,22^.

Interestingly, our cLBP group had increased MF, MPF, and lower values for MFs and MPFs. MF and MPF values for the EMG signals on the painful side also increased during the MVIC task. These results suggest that activity of the DM decreases with chronic pain whereas muscle activity usually increases with pain. These findings also support the idea that pain alters motor control patterns causing a redistribution of activation within a given muscle in a task specific manner^23^. Our findings also demonstrated that neuromuscular activation of the DM increases with persistent pain during MVIC. Considering that our cLBP patients were mainly young and middle-aged adults, changes in frequency domain may be compensatory effects to equalize atrophied deep LM muscles in the early stages of disease. Moreover, subjects with cLBP with a BMI ≤24 were used to ensure optimal EMG recordings and avoid the adipose tissue signals affect such as frequency and amplitude. Our cLBP patients also had a greater sensitivity to pain, possibly suggesting that chronic back pain alters spontaneous neuronal activity resulting in muscle performance and EMG activity changes.

Consistent with previous research reporting significantly reduced muscle thickness and size of the DM in response to chronic pain stimuli^16,24^, our findings showed that cLBP patients had a non-specific global reduction in DM resting thickness, cross-sectional area and thickness during MVIC measured by ultrasound. Although some studies report significantly more atrophy in the DM on the painful side^11,12^, we found no side-to-side differences in this study. The fact that differences were observed for global reduction and not unilateral atrophy supports not only task specificity, but also a movement direction specific effect of pain on DM activity. Our results are similar to the findings of Sweeney *et al*.^14^ and Dickx *et al*.^16^ who found that induced unilateral LBP had no side-specific effect on DM activation, suggesting that unilateral pain may have a general effect on DM activation during MVIC movement. Also, reduced DM activation during maximum voluntary contraction was seen in the cLBP group with lower percentage change in DM thickness. This difference was statistically significant at the L4 vertebral level, suggesting that subjects with cLBP develop maladaptive movement and motor control impairments^25^.

Understanding the relationship between functional and morphological change in DM in cLBP can help provide insight into the mechanisms underlying motor control of the lumbar spine in response to nociceptive stimulation of low back muscles. Patients with cLBP had a decreased AEMG and increased MF, indicating that pain leads to reduced ability to voluntarily recruit the DM during functional tasks. AEMG in our cLBP group was positively correlated with contractile activity and not correlated with noncontractile anatomical abnormalities indicating that altered activity of the DM may be dampened secondary to pain. Our findings showed that muscular atrophy and pain duration were highly correlated, supporting the hypothesis that structural change is primarily related to painful stimuli. This change potentially reduces the myogenic support of the deep lumbar musculature and may lead to increased activity of more superficial muscles to stabilize the lumbar spine^19^. Also, findings on the magnitude of muscle damage have distinct associations with VAS scores, suggesting a strong link between impaired contractile functions and pain intensity^26^.

This study using the sEMG system combined with fine-wire electrodes to detect EMG signals of the DM provides a new method for future research to identify neuromuscular activation patterns of the deep muscles in different functional movements. However, there are some limitations to this methodology. First, the method of fine-wire EMG produced mild pain/discomfort caused by the insertion of needles. Second, as with previous studies that show MVIC elicits pain in cLBP populations, individuals may not perform their “maximum” ability during isometric muscle actions. This could be due to changes in muscle activation. To reduce this effect, verbal encouragement was given in the present study to achieve maximum contraction to produce a better outcome. Moreover, the potential for this “integrated” sEMG system with fine-wire EMG has not been explored as a potential model for other studies. Therefore, future studies are needed to assess the reliability and validity of this method for evaluating the inherent contraction properties of deep muscles.

In conclusion, our findings showed that cLBP is associated with abnormal EMG activity detected by the sEMG system and morphological features seen on ultrasounds of the DM at the L4 level. Our results suggest a relationship between AEMG and contractile elements during pain-processing in cLBP. Our correlational analyses provide initial data on DM activation during MVIC to clarify the mechanisms underlying motor control impairment of the back muscle in patients with unilateral cLBP. These analyses also show that differences may exist in the contractile behavior of the DM during functional movement. Development of a tool to assess the functional changes in the DM involved in lumbar stability as they relate to outcomes of conservative treatment as part of the clinical assessment for patients with cLBP is needed. Future studies should assess larger cohorts, consider gender-related factors, and sub-classify differences to investigate motor control patterns and muscle contractile dynamics in cLBP.
